# Biochemical and developmental characterization of carbonic anhydrase II from chicken erythrocytes

**DOI:** 10.1186/1751-0147-53-16

**Published:** 2011-03-07

**Authors:** Toshiho Nishita, Yuichiro Tomita, Takao Imanari, Nobutsune Ichihara, Kensuke Orito, Kazuyoshi Arishima

**Affiliations:** 1 Laboratories of Veterinary Physiology 1, School of Veterinary Medicine, Azabu University,1-17-71 Fuchinobe, Sagamihara, Kanagawa, 252-5201, Japan; 2 Isogaya Yokeien, Kamiishigami, Otawara-shi, Tochigi-ken, 324-0037, Japan; 3 Veterinary Anatomy 1, School of Veterinary Medicine, Azabu University,1-17-71 Fuchinobe, Sagamihara, Kanagawa, 252-5201, Japan; 4 Laboratories of Veterinary Physiology 2, School of Veterinary Medicine, Azabu University,1-17-71 Fuchinobe, Sagamihara, Kanagawa, 252-5201, Japan; 5 Veterinary Anatomy 2, School of Veterinary Medicine, Azabu University,1-17-71 Fuchinobe, Sagamihara, Kanagawa, 252-5201, Japan

## Abstract

**Background:**

Carbonic anhydrase (CA) of the chicken has attracted attention for a long time because it has an important role in the eggshell formation. The developmental profile of CA-II isozyme levels in chicken erythrocytes has not been determined or reported. Furthermore, the relations with CA-II in erythrocyte and egg production are not discussed. In the present study, we isolated CA-II from erythrocytes of chickens and determined age-related changes of CA-II levels in erythrocytes.

**Methods:**

Chicken CA-II was purified by a combination of column chromatography. The levels of CA-II in the hemolysate of the chicken were determined using the ELISA system in blood samples from 279 female chickens, ages 1 to 93 weeks, 69 male chickens, ages 3 to 59 weeks and 52 weeks female Araucana-chickens.

**Results:**

The mean concentration of CA-II in hemolysate from 1-week-old female was 50.8 ± 11.9 mg/g of Hb. The mean levels of CA-II in 25-week-old (188.1 ± 82.6 mg/g of Hb), 31-week-old (193.6 ± 69.7 mg/g of Hb) and 49-week-old (203.8 ± 123.5 mg/g of Hb) female-chickens showed the highest level of CA-II. The levels of CA-II in female WL-chickens significantly decreased at 63 week (139.0 ± 19.3 mg/g of Hb). The levels of CA-II in female WL-chicken did not change from week 63 until week 93.The mean level of CA-II in hemolysate of 3-week-old male WL-chickens was 78.3 ± 20.7 mg/g of Hb. The levels of CA-II in male WL-chickens did not show changes in the week 3 to week 59 timeframe. The mean level of CA-II in 53-week-old female Araucana-chickens was 23.4 ± 1.78 mg/g of Hb. These levels of CA-II were about 11% of those of 49-week-old female WL-chickens. Simple linear regression analysis showed significant associations between the level of CA-II and egg laying rate from 16 week-old at 63 week-old WL-chicken (p < 0.01).

**Conclusions:**

Developmental changes and sexual differences of CA-II concentration in WL-chicken erythrocytes were observed. The concentration of CA-II in the erythrocyte of WL-chicken was much higher than that in Araucana-chicken (p < 0.01).

## Background

Carbonic anhydrase (CA; EC 4.2.1.1), a well-characterized enzyme, catalyzes the reversible hydration of CO_2 _to form HCO_3_^- ^and protons according to the following reaction: CO_2 _+ H_2_O ↔ H_2_CO_3 _↔ HCO_3_^- ^+ H^+^. The first reaction is catalyzed by Carbonic anhydrase (CA) and the second reaction occurs instantaneously. CA plays important roles in gas transport, acid/base regulation, bone resorption and calcification, ion transport, and various secretary functions in several tissues [1]. There are at least 16 different carbonic anhydrase isozymes, each with a unique molecular structure. CA-I and CA-II, the main CA isozymes in human erythrocytes, are immunologically distinct [2]. Although CA-II has 30-fold higher enzymatic activity than CA-I, it accounts for only 11% of the total erythrocyte CA activity in humans.

CA of the chicken has attracted attention for a long time because it has an important role in the eggshell formation. Large amounts of Ca^2+ ^and CO_3_^2- ^are ultimately deposited as a CaCO_3 _shell on the egg. Gutowska and Mitchell [3] reported that the deposition of the CaCO_3 _occurs mainly in the shell gland of birds, and the source of the CO_3_^2- ^was thought to be the HCO_3_^- ^in blood. However, Hodges and Lorcher [4] reported that the source of the CO_3_^2- ^is not circulatory HCO_3_^- ^from the blood and that the shell gland derives CO_3_^2- ^from its own metabolic CO_2 _production. Pearson et al. [5] reported that the CA activity of uteri from laying quail is twice that of uteri from molting quail and five times greater than that of uteri from nonlaying quail. They suggested that the activity of CA in quail uterus may be related to uterine calcium secretion.

A single high activity form of the enzyme, homologous to mammalian CA-II, has been isolated from chicken erythrocytes [6]. Baumann et al. [7] reported that CA activity of primitive red cells in normoxic embryos declines from Day 4 to Day 6 and the minimum activity was found in 8- to 12-day-old embryos. And then, there was a sharp rise of CA activity from Day 14 at Day 18.

To our knowledge, the developmental profile of CA-II levels in chicken erythrocytes has not been determined or reported. Furthermore, the relations with CA-II in erythrocyte and egg production are not discussed. Thus, we determined the levels of CA-II in erythrocytes of male and female White Leghorn (WL)-chickens which have a high rate of egg production as they aged. The levels of CA-II in female Araucana-chickens, a low rate egg production, were measured to compare WL-chickens.

## Methods

### Blood samples for purification

White leghorn hens (LOHMAN LSL-LITE) were anesthetized with nembutal and a blood sample was taken from the heart. The blood was collected into a bottle containing 3.8% citric acid. All experiments were performed according to the guidelines of The Laboratory Animal Care Committee of Azabu University and complied with the Japanese Animal Welfare Guide.

### Purification of CA-II

**Figure 1:**
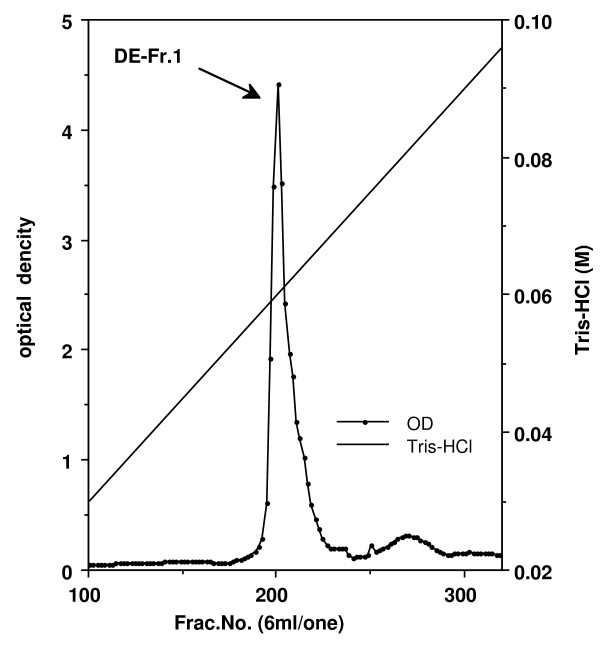
**Chromatogram illustrating the separation of chicken carbonic anhydrase on a DEAE-cellulose column when using Tris-HCl (pH 8.0) from 0.001 M to 0.1 M as an elution buffer**. Optical density of each fraction was determined at 280 nm. Column size: 3.4 × 30 cm.

**Figure 2:**
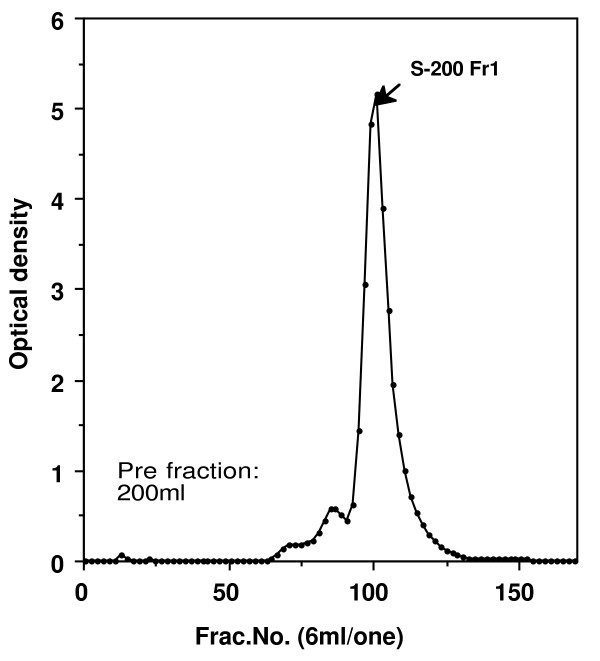
**Results of Sephacryl S-200 HR column gel filtration experiment on the DE-Fr1 fractions shown in figure 1**. Column size: 3.4 × 30 cm. Starting buffer: 0.05 M Tris-HCl (pH 8.0) containing 0.5 M NaCl. Flow rate: 20 mL/h.

**Figure 3:**
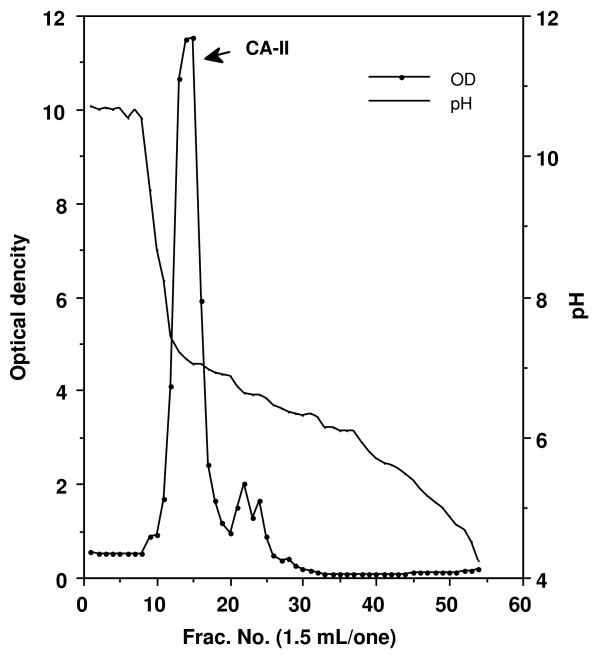
**Results of column electrofocusing of the fractions that constituted the S200 Fr1 peak in figure 2**. Values were obtained by use of a spectroscope at 280 nm. OD = Optical density. Ampholine: pH5.0-7.0.

The pooled erythrocytes were washed with four volumes of 0.15 M NaCl and centrifuged at 1,400 × g for 30 min. This wash procedure was repeated two additional times. Packed erythrocytes were hemolyzed with an equal volume of distilled water. The hemolysates were then centrifuged at 27,000 × g for 30 min (at 4°C), and the pellet containing the stroma was removed. This supernatant was then alkylated by adding iodoacetamide to 0.01 M, the pH being adjusted to 8.0. Hemoglobin (Hb) was extracted using the chloroform/ethanol denaturation method (Tuchihasi extract), previously used for preparation of a number of other mammalian carbonic anhydrase [8]. First, 3.5 g NaCl was added to 400 ml of hemolysate. After cooling to 0°C in an ice bath, 85 ml of cold (0-5°C) 95% ethanol was slowly added into the hemolysate while the solution was stirred. Next, 105 ml of cold (0-5°C) chloroform was added. After 15 min, the precipitated hemoglobin was removed by centrifugation at 27,000 × g for 30 min at 0°C. The supernatant solution containing CA activity was dialyzed against 0.001 M Tris-HCl (pH 8.0) at 4°C. The dialyzed material was centrifuged and the supernatant was applied to a column (3.4 × 30 cm) of DEAE-cellulose (Whatman International Ltd., Maidstone, England) equilibrated with the 0.001 M Tris-HCl (pH 8.0) at 4°C. After washing the column thoroughly, the adsorbed proteins were eluted with a linear gradient of Tris-HCl (pH 8.0) between 0.001 and 0.1 M, and the optical densities of all fractions were read at 280 nm (Figure 1). The fractions that contained enzyme activity were collected and pooled. The enzymatic activities of pooled samples were detected by the method of Wilbur and Anderson [9], and the samples were dialyzed against saturated ammonium sulfate. The precipitates were collected by centrifugation. The precipitates were dissolved in a small amount of distilled water and dialyzed completely against 0.01 M Tris-HCl (pH 8.0) at 4°C. Next, samples were passed over a Sephacryl S-200 HR (Sephacryl HR, Pharmacia Biotech, Uppsala, Sweden) column equilibrated with 0.05 M Tris-HCl (pH 8.0) containing 0.5 M NaCl, at a flow rate of 20 ml/h. The fractions that contained enzyme activity were collected and pooled (Figure 2). Samples were dialyzed against water and further purified using the column electrofocusing method of Svenson [10] using the LKB 8100 Ampholine electrofocusing column and Ampholine (pH 5.0-7.0) (Pharmacia Biotech). The fractions that contained enzyme activity were collected (Figure 3) and dialyzed against 0.01 M Tris-HCl buffer (pH 7.5). Purified chicken CA-II was stored at -80°C until use.

### Electrophoretic procedures

SDS-PAGE by the method of Laemmli [11] was performed on 12.5% homogeneous polyacrylamide gels (PhastGel separation media) using PhastSystem (Pharmacia Biotech).

Thin-layer isoelectric focusing was performed using PhastGel IEF media and the PhastSystem.

A low molecular weight calibration kit and a pI calibration kit (Pharmacia Biotech) were used to determine the molecular weight and the isoelectric point of the purified CA-II. The low molecular weight kit contains phosphorylase (94 kD), albumin (67 kD), ovalbumin (43 kD), carbonic anhydrase (30 kD), trypsin inhibitor (20.1 kD), and alpha-lactalbumin (14.4 kD) as standards. The low pI kit contains glucose oxidase (4.15), soybean trypsin inhibitor (4.55), beta-lactoglobulin A (5.20), bovine carbonic anhydrase B (5.85), and human carbonic anhydrase B (6.55). The high pI kit contains beta-lactoglobulin A (5.20), bovine carbonic anhydrase B (5.85), human carbonic anhydrase B (6.55), horse myoglobin-acidic band (6.85), horse myoglobin-basic band (7.35), lentil lectin-acidic band (8.15), lentil lectin-middle band (8.45), lentil lectin-basic band (8.65), and trypsinogen (9.30).

### Enzyme assay

Enzymatic activity of CA in the hemolysate or purified CA-II was measured using the method of Wilbur and Anderson [9]. Assays were performed at 4°C, and specific activity (U) was determined according to the formula: U = 10 × (Tb/Te-1)/mg of protein

Where Tb is the time required for the uncatalyzed reaction (i.e., pH change from 8.5 to 6.5), and Te is the time required for the enzyme-catalyzed reaction. The reaction time was measured with a stopwatch.

### Antiserum

Antibodies against purified chicken CA-II were produced in rabbits. Each rabbit was injected initially with 1 mg of purified CA-II emulsified with an equal volume of Freund's complete adjuvant, followed by a booster injection of an equivalent amount of purified CA-II once a week for five successive weeks. Rabbits were bled through the auricular vein 10 days after the last injection. The specificity of the antiserum was examined by a double immunodiffusion method. Immunoglobulin G (Ig G) was purified using Protein A.

### Biotinylation of chicken CA-II

A 2-ml volume of solution containing purified chicken CA-II (5 mg/ml) was incubated for 4 h at 25°C with 4.54 mg of biotin (sulfosuccinimidyl N-(N = -(D-biotinyl)-6-aminohexanoyl)-6-aminohexanoate) (Dojindo Laboratories, Kumamoto, Japan) in 0.08 ml of 10 mM HEPES buffer (pH 8.5). Conjugates were then dialyzed extensively against PBS (pH 7.5).

### Blood samples

Blood samples of clinically normal male WL-chickens (SPF, Line-M) (3 weeks old (n = 9), 10 weeks old (n = 10), 12 weeks old (n = 10), 17 weeks old (n = 10), 20 weeks old (n = 10), 27 weeks old (n = 5), 30 weeks old (n = 5), 52 weeks old (n = 5), and 59 weeks old (n = 5)) were purchased from Nisseiken Co., Ltd., (Tokyo, Japan). Blood samples of clinically normal female WL-chickens (LOHMAN LSL-LITE) (1 week old (n = 20), 3 weeks old (n = 18), 9 weeks old (n = 19), 12 weeks old (n = 20), 16 weeks old (n = 20), 21 weeks old (n = 20), 25 weeks old (n = 20), 31 weeks old (n = 20), 49 weeks old (n = 19), 63 weeks old (n = 20), 69 weeks old (n = 20), 73 weeks old (n = 20), 80 weeks old (n = 20), and 93 weeks old (n = 20)) were provided by Isogaya Yokeien (Tochigi, Japan). The forced molt was induced at 64 weeks old and finished at 67 weeks old. Blood samples of laying female Araucana-chickens, 53 weeks old (n = 6), were provided by Kanagawa Prefectural Livestock Industry Technology Center (Kanagawa, Japan). Blood samples were mixed with lithium heparin and centrifuged at 4,500 × g for 15 min (at 4°C) to separate plasma from erythrocytes. Erythrocytes were hemolyzed with equal volume of distilled water and centrifuged at 27,000 × g for 30 min (at 4°C). Hemolysates were stored at -20° C until analyzed the concentration of CA-II. Hemolysate samples of 0.1 ml, diluted at 1/4,000 to 1/16,000 with 50 mM Tris-HCl (pH 7.5) containing 0.3% BSA, 0.9% NaCl, 0.01% thimerosal, and 10 mM EDTA (buffer A), were subjected in duplicate to ELISA.

The egg laying rate of WL-chicken at the ages of 21, 25, 31, 49, 63, 69, 73, 80 and 93 weeks old, were about 20, 94, 94, 95, 91, 2, 63, 91 and 82%, respectively. At the ages of 1, 3, 9, 12 and 16 weeks old, the egg-laying rate of WL-chicken was 0%. The average egg-laying rate of Araucana-chicken at 53 weeks old was about 65%.

### Determination of CA-II concentration

The concentrations of CA-II in chicken erythrocytes were determined using a competitive ELISA method. A flat-bottom micro-ELISA plate (Nunc immuno-plate, Maxisorp, Roskilde, Denmark) was coated for 16 h at 4°C with 0.1 ml/well of anti-chicken CA-II IgG dissolved in 0.1 M NaHCO_3 _(pH 8.5). Plates were then washed three times with 0.3 ml/well PBS and incubated at 23°C for 1 h with 0.2 ml/well of 0.5% BSA in 0.05 M Tris-HCl (pH 8.0) for blocking. Next, each well was washed three times with 0.3 ml/well 0.5% Tween in PBS (PBS-Tween). Each standard CA-II sample (2-800 ng/ml), biotinylated chicken-CA-II sample, and chicken hemolysate was diluted with buffer A, and duplicate ELISAs were performed. The biotinylated CA-II put in competition with the standard CA-II or with CA-II in the hemolysate and incubation for 16 h at 4°C. And then, each well was washed with PBS-Tween three times, and 0.1 ml/well of avidin and biotinylated horseradish peroxidase complex (ABC reagent: Wako Pure Chemical Industries Ltd, Tokyo, Japan) was added. ABC reagent was diluted 1:100 with PBS-Tween. After 30 min, each well was washed three times with PBS-Tween. Peroxidase activity was measured after addition of 0.1 ml/well ABTS microwell peroxidase substrate system (Kirkegaard & Perry Laboratories Inc, Gaithersburg, CA, USA). The ABTS substrate system contained 2,2'-azino-di-(3-ethylbenzthiazoline-6-sulfonate) at a concentration of 0.3 g/L in a glycine/citric acid buffer. The concentration of the H_2_O_2 _was 0.01%. After 10 min, 0.1 ml/well of 1% sodium dodecyl sulfate was added to terminate the enzyme reaction and the absorbance was read on an automatic ELISA reader at 405 nm (SH-1000: Corona Electric Co., Ltd., Ibaraki, Japan).

### Optimization of the ELISA system

To determine optimum assay conditions, several experiments were performed. First, the microplate was coated with several concentrations of antibody and calibration curves were drawn. A concentration of 10 mg/ml of antibody was chosen as the first standard for the assay, and the concentration of 0.01 mg/ml biotinylated chicken CA-II was also chosen. Assay precision was evaluated using standard samples of 400, 200, 100, 50, 25, 12, and 6 ng/ml of purified CA-II, and five replicates of each concentration were assayed one assay run. The coefficients of variation for each assay were less than 5%. To test the sensitivity of the assay, the hemolysate was diluted to 1/8,000, 1/16,000, and 1/20,000 with buffer A. A linear relationship between estimation of CA-II concentration and dilution of the hemolysate was obtained from 1/4,000 to 1/16,000.

### Hemoglobin assay

The hemoglobin concentration in the hemolysate was measured by the sodium lauryl sulfate hemoglobin method using hemoglobin B testowako (Wako Pure Chemical Industries Ltd.).

### Protein assay

The protein concentrations of the aliquots solution of purified chicken CA-II were determined using a DC Protein Assay kit (Bio-Rad Laboratories, Hercules, CA, USA).

### Statistical analysis

Values are expressed as means ± SD. Statistical differences in the levels of CA-II of male and female chicken erythrocytes were analyzed using Student's t-test. Statistical differences in the level of CA-II of each age group were evaluated using a one-way analysis of variance (ANOVA) followed by the Bonferroni post-hoc test. Simple linear regression analysis was used to estimate the relationship between the level of CAII and egg laying percentage. A significance level of P < 0.05 was chosen.

## Results

### Purification and properties of chicken CA-II

The elution profiles for each of the purification steps are shown in Figure 1, 2, and 3. Fractions from the first peak on the DEAE-cellulose chromatograph (DE-Fr1) contained CA activity (Figure 1). These fractions were passed over a Sephacryl S-200 HR column (Figure 2), and fractions from the main peak of the S-200 column contained CA activity. The results of column isoelectrofocusing are shown in Figure 3. The fractions from the main isoelectrofocusing peak contained CA activity.

**Figure 4:**
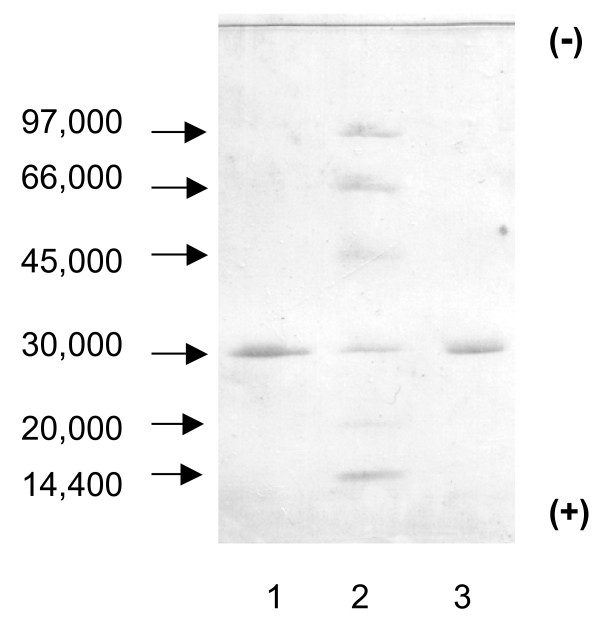
**Results of SDS-PAGE of purified CA isozymes**. Lane 1; Dog CA-II, Lane2; Molecular weight marker. The low molecular weight kit contains phosphorylase (94 kD), alubumin (67 kD), ovalubumin (43 kD), carbonic anhydrase (30 kD), trypsin inhibitor (20 kD) and alpha-lactalbumin (14,4 kD)., Lane 3; Chicken CA-II.

**Figure 5:**
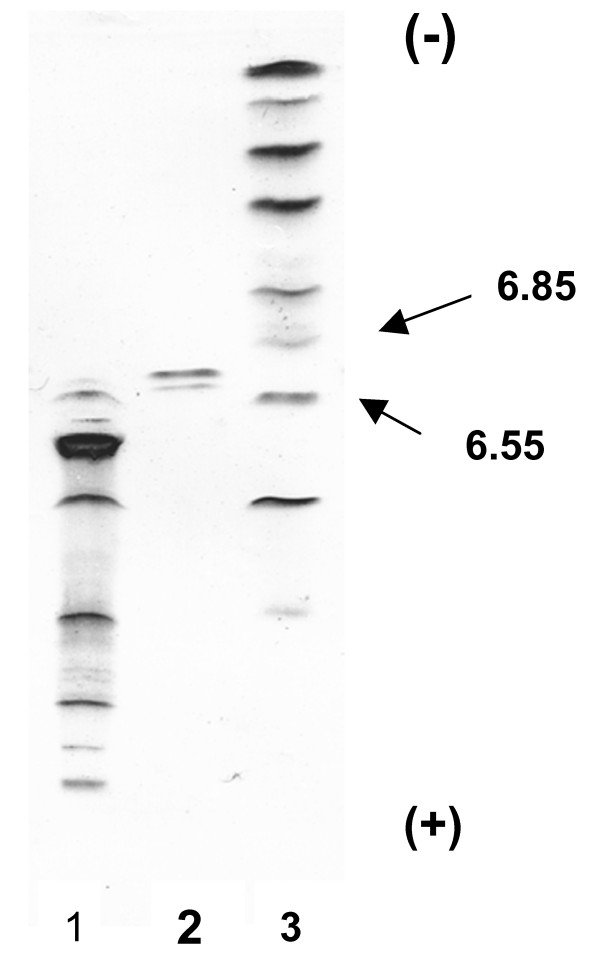
**IEF-PAGE of purified chicken CA-II**. Lane 1; Low pI calibration marker (pI 2.5-6.5). Lane 2; Chicken CA-II Lane 3; High pI calibration marker (pI 5.2-10.3).

The CA purified from chicken erythrocytes resolved into one band by SDS-PAGE and had an estimated molecular weight of 30,000 (Figure 4). Canine CA-II, which was purified in our laboratory [12], had an estimated molecular weight similar to that of CA purified from chicken erythrocytes. A thin-layer isoelectric focusing polyacrylamide gel electrophoretogram of CA purified from chicken erythrocytes is shown in Figure 5. Isoelectric points of 6.7 and 6.6 were observed for the purified chicken CA.

### Enzyme activity

The specific activity of the CA-II purified from chicken erythrocytes, as measured using the method of Wilbur and Anderson [9], was 19,000 units/mg protein.

#### Specificity of chicken CA-II antiserum

The specificity of antiserum against chicken CA-II produced in rabbits was evaluated by double immunodiffusion (Ouchtalony method). Antiserum to chicken CA-II was reacted with chicken hemolysate and purified chicken CA-II with completely fused. Antiserum against chicken CA-II reacted with chicken CA-II, but not canine CA-II and CA-I (data not shown).

### Measurement of CA-II

**Figure 6:**
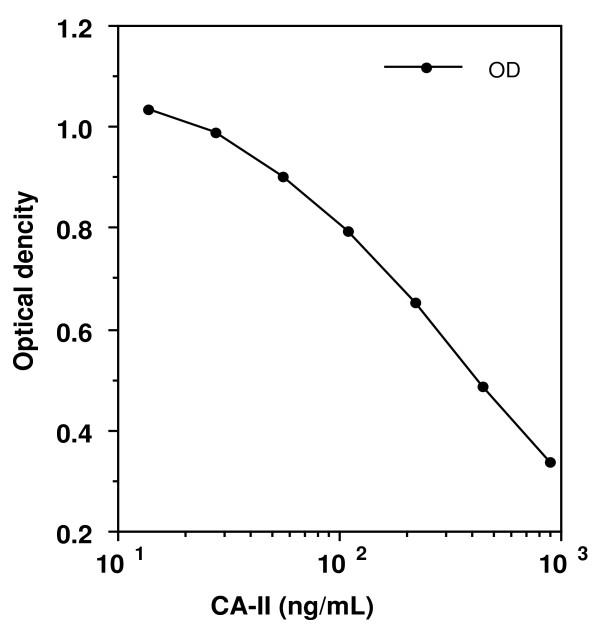
**Standard curves for chicken CA-II concentrations generated by use of an ELISA specific for chicken CA-II**. Values were obtained by use of a spectroscope at 405 nm.

**Figure 7:**
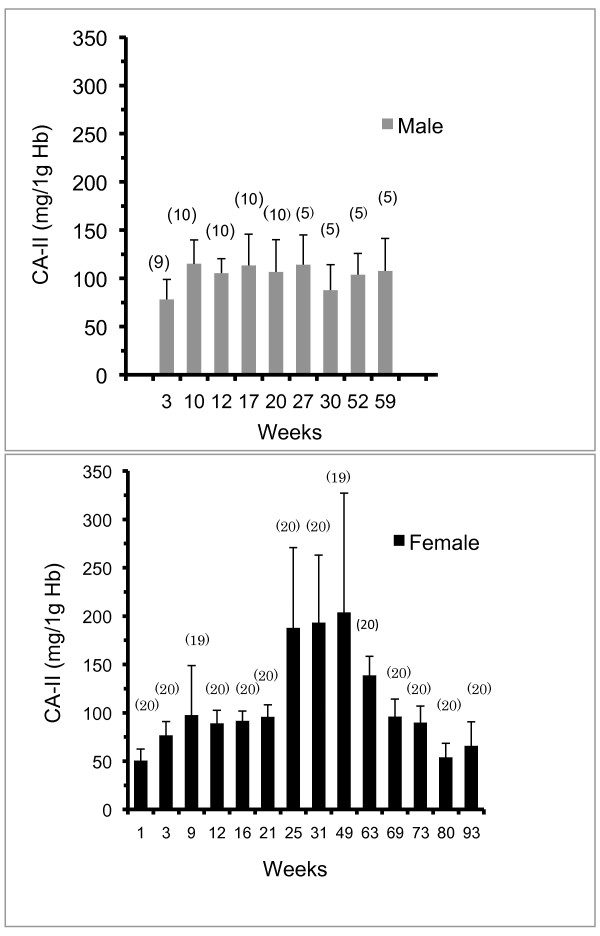
**Developmental changes of CA-II in male and female WL-chicken erythrocytes**. Mean and standard deviation of CA-II concentration (mg/g of Hb) of erythrocytes from male (top) and female (bottom) WL-chicken. The number of samples is shown on the column.

Typical standard curve for a reference range of CA-II solutions (20-800 ng/ml) is shown in Figure 6. The concentrations of CA-II in hemolysate samples from female WL-chickens (1 to 93 weeks old) and male WL-chickens (3 to 59 weeks old) were assayed using competitive ELISA and the results (mean ± SD) of developmental changes are shown in Figure 7. The mean concentration of CA-II in hemolysate from 1-week-old female WL-chickens was 50.8 ± 11.9 mg/g of Hb, and then CA-II levels did not change until week 21. The levels of CA-II in female WL-chickens increased significantly at 25, 31 and 49 week (P < 0.01). The mean levels of CA-II in hemolysate from 25-week-old (188.1 ± 82.6 mg/g of Hb), 31-week-old (193.6 ± 69.7 mg/g of Hb) and 49-week-old (203.8 ± 123.5 mg/g of Hb) female WL-chickens showed the highest level of CA-II. The levels of CA-II in female WL-chickens decreased significantly at 63 week (139.0 ± 19.3 mg/g of Hb) (p < 0.01). The levels of CA-II in female WL-chicken decreased gradually from weeks 69 until weeks 93. Simple linear regression analysis showed significant associations between the level of CA-II and egg laying rate from 16 week-old at 63 week-old WL-chicken (p < 0.01). On the other hand, the associations between the egg laying rate and CA-II levels of in the erythrocytes from 69 week-old until 93 week-old was not observed.

The mean level of CA-II in hemolysate of 3-week-old male WL-chickens was 78.3 ± 20.7 mg/g of Hb. The levels of CA-II in hemolysate of male WL-chickens did not show statistically significant changes in the week 3 to week 59 timeframe.

There was a difference (p < 0.05) of CA-II level between 12, 16, 25, 31, 49 and 63 week-old female WL-chickens and 12, 17, 27, 30, 52 and 59 week-old male WL-chickens. The mean level of CA-II in hemolysate of 53-week-old female Araucana-chickens was 23.4 ± 1.78 mg/g of Hb. These levels of CA-II were about 11% of those of 49-week-old female WL-chickens and about 25% of those of 52-week-old male WL-chickens. There was a significant difference of CA-II level between 53 week-old female Araucana-chicken and 49 and 63 week-old female WL-chickens (p < 0.01).

## Discussion

### Identification of Chicken CA-II

CA-I and CA-II are both found in the erythrocytes of most mammalian species with the exception of ruminants and felines, which have only CA-II isozyme in erythrocytes [13,14]. The specific activities of equine CA-I and CA-II are 3,400 units/mg protein and 36,000 units/mg protein, respectively [15], and Holmes [16] reported that specific activity of chicken CA-II was 20,300 units/mg protein. Furthermore, CA-I was purified from intestine, and the specific activities of the chicken CA isozymes differed significantly with CA-II being 7.8-fold more active than CA-I. The reported specific activities of equine and chicken CA-II are similar to 19,000 units/mg protein, the specific activity of CA purified from chicken erythrocytes, and taken together these findings are consistent with the conclusion that the high activity CA isozyme from purified from chicken erythrocytes in the present study is CA-II.

Bernstein and Schraer [6] purified high activity CA from the erythrocytes of White Leghorns, apparently CA-II, using dithiothreitol (DTT) during purification. They also reported that chicken CA-II contains 7 moles of half-cystine per mole of enzyme. They suggested that CA-II may exist in the fully reduced state under physiological conditions in chicken erythrocytes because maintaining enzyme in vitro requires reducing reagents such as DTT or 2-mercapoethanol. Therefore, we used iodoacetamide to prevent disulfide bonds from forming between cystine residues in the purified CA-II in the present study. The only other animal CA-II which contains more than a single half-cystine residue is the equine CA-II, which contains 2 cystine residues [17]. Mammalian CA-III, which has two sulfhydryl groups, may have a role in scavenging oxygen radicals and, therefore, protecting cells from oxidative damage [18,19]. Based on this structural similarity between chicken CA-II and mammalian CA-III, it is interesting to examine further about a role of chicken CA-II in scavenging of oxygen radicals.

Holmes [16] reported that a zymogram of CA isozymes (CA-II) from chicken red blood cells revealed 3 forms that were widely distributed throughout the body. Grunder and Hollands [20] reported that starch gel electrophoretic patterns of erythrocytes disclosed 9 zones of CA isozymes, and no qualitative differences were found in enzyme patterns between birds laying high specific gravity eggs and those laying low specific gravity eggs. There have been a number of previous reports of electrophoretically distinct zones of CA-I and CA-II. The multiplicity of CA isozymes was subsequently shown to result from an epigenetic modification of CA-I, which resulted from the loss of amide groups [21]. In the present study, the band at isoelectric point of 6.6 may represent chicken CA-II (PI: 6.7) that has lost amide groups.

### The levels of CA-II in erythrocytes

Kondo et al. [22] reported that the concentration of CA-II in human erythrocytes was approximately 3.2 mg/g of Hb and that of CA-I was approximately 14 mg/g of Hb. Although little variation in the levels of CA isozymes in erythrocytes was observed within the healthy human population, a wide variation occurs in various disease states [2]. Furthermore, an increase of almost 20% above baseline values for both CA-I and CA-II was observed in the maternal erythrocytes at parturition [23]. The levels of CA-I and CA-II in erythrocytes of Beagles were 3.21 and 1.63 mg/g of Hb, respectively. In pregnant Beagles, there was an increase in CA-II of approximately 15% above that of non-pregnant female Beagles and 30% above that of male Beagles [12]. Mean concentrations of CA-I and CA-II in racehorses were 1.70 and 0.94 mg/g of Hb, respectively [15]. In the present study, mean concentration of CA-II in erythrocytes of non-laying WL-chickens (i.e., 21 weeks old) was 96.1 ± 12.2 mg/g of Hb, and it was 59 times of the levels of CA-II in erythrocytes of Beagles. The mean level of CA-II in hemolysate of 53-week-old female Araucana-chickens was 23.4 ± 1.78 mg/g of Hb. It was about 11% of those of 49-week-old female WL-chickens, about 25% of those of 52-week-old male WL-chickens and 14 times of the levels of CA-II in erythrocytes of Beagles. The levels of CA-II in erythrocytes of 53-week-old female Araucana-chickens was about the same levels to the value that added quantity of CA-I and CA-II in erythrocytes of human. These data mean that there is a clear difference in the level of CA-II between species and even between different lines of chickens. The levels of Hb in chicken, horse and dog were 7.0 ~13.0 g/dL, 11.0 ~ 19.0 g/dL and 12~18 g/dL, respectively. The erythrocyte count of chicken, horse and dog were 2.5~3.5 × 10^6^/μL, 6.8~12.9 × 10^6^/μL and 5.5~8.5 × 10^6^/μL, respectively [24]. From these data, the levels of chicken CA-II in erythrocytes were apparently much higher than those observed in mammals and may result in higher H_2_CO_3 _content. The maximum life span of the chicken erythrocyte was about 35 days [24]. It was thought that the high metabolic activity and high body temperature of the bird shortens the life span of erythrocyte. The high levels of CA-II in chicken erythrocytes were thought to have resulted from these metabolic characteristics of chickens.

When molecular CO_2 _diffuses into the erythrocyte, it is hydrated by CA-II and a bicarbonate ion and a proton are formed. The excess protons cause the intracellular pH to drop, and hemoglobin buffers the free protons. When hemoglobin binds a proton, its affinity for O_2 _decreases (the Bohr effect), and O_2 _is released from the hemoglobin, which is taken up at the tissues. The bicarbonate ion is then shuttled out of the erythrocyte. Thus CA-II supplies the HCO_3_^- ^substrate for transport and removes HCO_3_^- ^following transport. Modulation of CA-II level therefore provides a means to regulate the rate of HCO_3_^- ^transport.

### Changes of CA-II concentrations during growth

Baumann et al. [7] reported that the change of CA activity in primitive red cells from normoxic and hypoxic (incubation in 13.5% O_2_) chick embryos. CA activity of primitive red cells from both normoxic and hypoxic embryo declines from Day 4 to Day 6 and there is no significantly different between 4 and 6 days of development. In normoxic embryos the minimum CA activity was found in primitive red cells from 8- to 12-day-old embryos. From Day 14 onward there is a sharp rise of CA activity at Day 18. On the other hand, in hypoxia CA activity was already significantly increased at Day 8 of incubation. They suggested that *P*O_2 _has a controlling influence on the timing of differentiation events of definitive embryonic red cells. Everaert et al. [25] reported that the protein abundance and the activity of CA-II in erythrocytes was significantly higher in CO_2_-exposed embryos compared to normal conditions. They described that chicken embryos adapt to CO_2 _during the second half of incubation by increasing CA-II expressions which may serve to "buffer " elevated CO_2 _levels in red blood cells.

To assess whether CA-II concentrations in the erythrocytes changed during posthatch growth, chicken erythrocytes were analyzed over many weeks of growth and development. Developmental changes and sexual differences of CA-II concentrations in WL-chicken erythrocytes were observed. The levels of CA-II in male erythrocytes were relatively stable from 3 weeks to 63 weeks of development. The levels of CA-II in erythrocytes of 1-week-old female chickens were low, and these levels slightly increased to double until 21 weeks of development. And then, CA-II levels were significantly increased until 49 weeks old. The levels of CA-II in the erythrocytes of laying females at 25, 31, and 49 weeks old were about four times greater than those in erythrocytes from non-laying chickens (1 week old). The highest level of CA-II in hemolysate at 25, 31 and 49 weeks old female WL-chicken was 569 mg/g of Hb. Therefore, the variation of the level of CA-II during these ages was big. The variation may be due to heterogeneity of the erythrocytes population. After 63 week-old, the levels of CA-II were gradually decreased until 93 week-old. It is thought that the decrease of CA-II from 63 week-old may be caused by aging.

The levels of CA-II in erythrocytes of 12 and 16 weeks old male chickens were higher than those in erythrocyte from 12 and 17 weeks old female. On the other hand, CA-II levels of 27, 30, 52 and 59 weeks old male chickens were lower than those in erythrocyte from 25,31, 49 and 63 weeks old female. These data indicated that CA-II levels in male erythrocytes were higher than those in immature female and CA-II in female erythrocytes was significantly increased during the egg-laying period.

### Egg production and CA-II levels

Hodges and Lorcher [4] reported that the source of the CO_3_^2- ^for eggshell formation is not circulatory HCO_3_^- ^in the blood. Simkiss [26,27] reported that the intracellular pH of the shell gland fell to a mean value of 6.53 during the first 8 h of calcification and then rose to 6.97 during the latter part of shell formation. The formation of the eggshell of the chicken involves the deposition of about 5 g of CaCO_3 _over a period of 20 h, which corresponds with a period of metabolic acidosis during which the blood pH falls from 7.53 to 7.41 and HCO_3_^- ^from 31.5 to 20.7 m-equiv/L. The acidosis is induced by the shell gland of the oviduct during the formation of the CO_3_^2-^. The main role of CA-II in the erythrocyte is to generate H^+ ^and HCO_3_^- ^during acid-base regulation.

The forced molt was induced at 64 weeks and stopped at 67 weeks. At the ages of 63, 69, 73, 80 and 93 weeks old, the rates of egg lay were about 91, 2, 63, 91 and 82%, respectively. The levels of CA-II in the female chicken did not change significantly from 63-week-old until 93 week-old. Although the rate of egg lay was 2% at 69 week-old, the CA-II levels of 69 week-old were similar to that of 63-week old female chicken. It means that the synthesis of CA-II in the erythrocyte at 69 week-old was not affected by the forced molt.

The average laying eggs rate of Araucana-chickens (53 week-old) used in this study was about 65% and the levels of CA-II were 23% of those of 73 week-old WL-chicken of which the rate of egg production was 63%.

In the present study, positive correlation was observed between the egg production rate and CA-II levels in the erythrocytes of laying WL-chickens from 16 week-old until 49 week-old. But, positive correlation was not observed between the egg production rate and CA-II levels in the erythrocytes from 63 week-old until 69 week-old WL-chicken.

Tanabe et al. [28] reported that positive correlations were observed between the egg production rate and luteinizing hormone (LH), progesterone, and testosterone concentrations in the chicken plasma. However, there was no evidence that these hormones stimulated the synthesis of the CA-II within the erythrocyte of laying chicken.

Further investigations of physiological functions of CA-II in the erythrocytes of chicken are needed.

## Conclusions

Developmental changes and sexual differences of CA-II concentrations in WL-chicken erythrocytes were observed. There was a correlation between the egg laying rate and high levels of CA-II in the erythrocytes from 16 week-old at 49 weeks old WL-chicken. But, the correlation between the egg laying rate and CA-II levels in the erythrocytes from 63 week-old until 93 week-old was not observed. The concentration of CA-II in the erythrocyte of WL-chicken was much higher than that in Araucana-chicken (p < 0.01).

## Competing interests

The authors declare that they have no competing interests.

## Authors' contributions

Design of sample collection: TN, NI and YT. Sample collection and processing: TN, NI and YT. Survey design and implementation: TN, YT. Survey data entry: TN and KO. Analysis: TN, KO and KA drafted the paper; the other authors helped writing the paper. All authors read and approved the final manuscript.
